# *N*′-[(1*E*)-1-(3,5-Dichloro-2-hy­droxy­phen­yl)propyl­idene]-4-meth­oxy­benzohydrazide monohydrate

**DOI:** 10.1107/S1600536810037293

**Published:** 2010-09-25

**Authors:** Chun-Hong He, Jian-Ping Zhang, Jian-Guo Chang

**Affiliations:** a Department of Material Science and Chemical Engineering, Taishan University, 271021 Taian, Shandong, People’s Republic of China

## Abstract

The title compound, C_17_H_16_Cl_2_N_2_O_3_·H_2_O, displays a *trans* conformation with respect to the C=N double bond. The dihedral angle between the two benzene rings is 30.77 (5)° and there is one intra­molecular N—H⋯O hydrogen bond. In the crystal, mol­ecules are linked by hydrogen bonding to the water molecules of crystallization, which acts as both an acceptor and a donor, into a three-dimensional network.

## Related literature

For further details of the chemistry of the title compound, see: Carcelli *et al.* (1995 ▶); Salem (1998 ▶). For a related structure, see: Chang & Ji (2007 ▶).
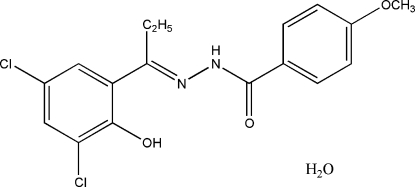


## Experimental

### 

#### Crystal data

C_17_H_16_Cl_2_N_2_O_3_·H_2_O
*M* *_r_* = 385.23
Monoclinic, 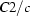

*a* = 32.925 (3) Å
*b* = 7.3733 (7) Å
*c* = 15.1252 (13) Å
β = 92.319 (2)°
*V* = 3668.9 (6) Å^3^
*Z* = 8
Mo *K*α radiation
μ = 0.38 mm^−1^
*T* = 295 K
0.21 × 0.16 × 0.11 mm

#### Data collection

Bruker APEXII CCD area-detector diffractometer
Absorption correction: multi-scan (*SADABS*; Sheldrick, 2003 ▶) *T*_min_ = 0.925, *T*_max_ = 0.960
9328 measured reflections
3242 independent reflections
2320 reflections with *I* > 2σ(*I*)
*R*_int_ = 0.027

#### Refinement

*R*[*F* ^2^ > 2σ(*F* ^2^)] = 0.042
*wR*(*F* ^2^) = 0.127
*S* = 1.01
3242 reflections
230 parameters
H-atom parameters constrained
Δρ_max_ = 0.29 e Å^−3^
Δρ_min_ = −0.39 e Å^−3^

### 

Data collection: *APEX2* (Bruker, 2005 ▶); cell refinement: *SAINT* (Bruker, 2005 ▶); data reduction: *SAINT*; program(s) used to solve structure: *SHELXS97* (Sheldrick, 2008 ▶); program(s) used to refine structure: *SHELXL97* (Sheldrick, 2008 ▶); molecular graphics: *SHELXTL* (Sheldrick, 2008 ▶); software used to prepare material for publication: *SHELXTL*.

## Supplementary Material

Crystal structure: contains datablocks global, I. DOI: 10.1107/S1600536810037293/fl2314sup1.cif

Structure factors: contains datablocks I. DOI: 10.1107/S1600536810037293/fl2314Isup2.hkl

Additional supplementary materials:  crystallographic information; 3D view; checkCIF report

## Figures and Tables

**Table 1:** Hydrogen-bond geometry (Å, °)

*D*—H⋯*A*	*D*—H	H⋯*A*	*D*⋯*A*	*D*—H⋯*A*
O1—H1⋯N1	0.82	1.77	2.494 (2)	146
N2—H2⋯O4^i^	0.86	2.16	3.004 (2)	168
O4—H18⋯O2^ii^	0.85	1.97	2.815 (2)	171
O4—H19⋯O1^iii^	0.85	2.11	2.908 (2)	155

Symmetry codes: (i) 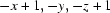; (ii) 
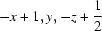
; (iii) 
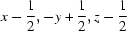
.

**Fig. 1.**
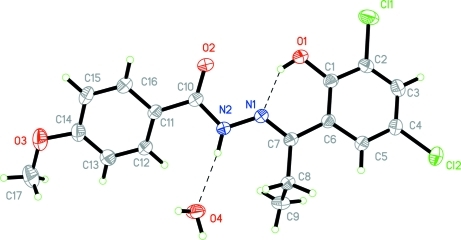
The molecular structure of compound (I), showing the atom-numbering scheme. Displacement ellipsoids are drawn at the 30% probability level.Dashed lines show intramolecular O–H···N hydrogen bonds.

**Fig. 2.**
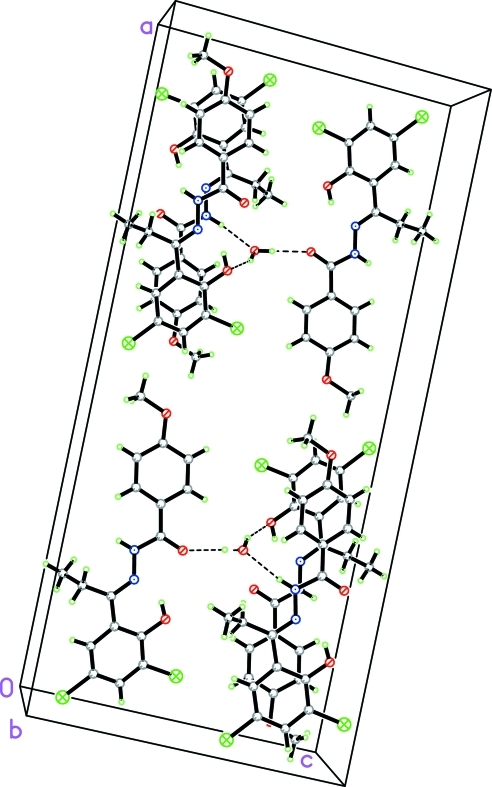
Packing diagram of (I), showing intermolecular O—H···O and N—H···O hydrogen bonds with the water molecule (dashed lines).

## supplementary crystallographic information

### Comment

The chemistry of aroylhydrazones continues to attract much attention due to their ability to coordinate with metal ions (Salem, 1998) and their biological activity (Carcelli *et al.*, 1995). As an extension of work on the structural characterization of aroylhydrazone derivatives (Chang & Ji, 2007), the title compound, (I),was synthesized and its crystal structure is reported here.

(I) displays a trans conformation with respect to the C7=N1 double bond (Fig. 1). The N2-N1=C7-C6 torsion angle is -175.44 (18)°.The dihedral angle between the two benzene rings is 30.77 (molecules are linked into a three dimensional network by intermolecular N-H···O, O-H···O Hydrogen bonds involving the water molecule which acts as both a donor and an acceptor.( Table. 1, Fig. 1and Fig. 2).

### Experimental

4-methoxybenzohydrazide (0.01 mol,1.66 g) was dissolved in anhydrous ethanol (40 ml), and 1-(3,5-dichloro-2-hydroxyphenyl)propan-1-one (0.01 mol, 2.19 g) was added. The reaction mixture was refluxed for 6 h with stirring, then the resulting precipitate was collected by filtration, washed several times with ethanol and dried *in vacuo* (yield 82%). The compound (1.0 mmol, 0.37 g) was dissolved in dimethylformamide (15 ml) and kept at room temperature for 30 d to obtain colourless single crystals suitable for X-ray diffraction.

### Refinement

All H atoms were positioned geometrically and treated as riding on their parent atoms,with *C*—*H*(methyl) = 0.96 Å, *C*—*H*(methylene) = 0.97 Å,*C*—H(aromatic) = 0.93 Å, O—H = 0.82 Å, *N*—H =0.86 Å and with *U*_iso_(H) =1.5*U*_eq_(C_methyl_,*O*) and 1.2*U*_eq_(C_aromatic_,*C*_methylene_,*N*).

### Crystal data

**Table:**

C_17_H_16_Cl_2_N_2_O_3_·H_2_O	*F*(000) = 1600
*M**_r_* = 385.23	*D*_x_ = 1.395 Mg m^−^^3^
Monoclinic, *C*2/*c*	Mo *K*α radiation, λ = 0.71073 Å
Hall symbol: -C 2yc	Cell parameters from 2261 reflections
*a* = 32.925 (3) Å	θ = 2.7–22.8°
*b* = 7.3733 (7) Å	µ = 0.38 mm^−^^1^
*c* = 15.1252 (13) Å	*T* = 295 K
β = 92.319 (2)°	Block, colourless
*V* = 3668.9 (6) Å^3^	0.21 × 0.16 × 0.11 mm
*Z* = 8	

### Data collection

**Table:**

Bruker APEXII CCD area-detector diffractometer	3242 independent reflections
Radiation source: fine-focus sealed tube	2320 reflections with *I* > 2σ(*I*)
graphite	*R*_int_ = 0.027
φ and ω scans	θ_max_ = 25.1°, θ_min_ = 2.5°
Absorption correction: multi-scan (*SADABS*; Sheldrick, 2003)	*h* = −38→31
*T*_min_ = 0.925, *T*_max_ = 0.960	*k* = −7→8
9328 measured reflections	*l* = −17→18

### Refinement

**Table:**

Refinement on *F*^2^	Secondary atom site location: difference Fourier map
Least-squares matrix: full	Hydrogen site location: inferred from neighbouring sites
*R*[*F*^2^ > 2σ(*F*^2^)] = 0.042	H-atom parameters constrained
*wR*(*F*^2^) = 0.127	*w* = 1/[σ^2^(*F*_o_^2^) + (0.0624*P*)^2^ + 2.1699*P*] where *P* = (*F*_o_^2^ + 2*F*_c_^2^)/3
*S* = 1.01	(Δ/σ)_max_ = 0.001
3242 reflections	Δρ_max_ = 0.28 e Å^−^^3^
230 parameters	Δρ_min_ = −0.39 e Å^−^^3^
0 restraints	Extinction correction: *SHELXL97* (Sheldrick, 2008)
Primary atom site location: structure-invariant direct methods	Extinction coefficient: 0.0014 (2)

### Special details

**Table:**

Geometry. All e.s.d.'s (except the e.s.d. in the dihedral angle between two l.s. planes) are estimated using the full covariance matrix. The cell e.s.d.'s are taken into account individually in the estimation of e.s.d.'s in distances, angles and torsion angles; correlations between e.s.d.'s in cell parameters are only used when they are defined by crystal symmetry. An approximate (isotropic) treatment of cell e.s.d.'s is used for estimating e.s.d.'s involving l.s. planes.
Refinement. Refinement of *F*^2^ against ALL reflections. The weighted *R*-factor *wR* and goodness of fit *S* are based on *F*^2^, conventional *R*-factors *R* are based on *F*, with *F* set to zero for negative *F*^2^. The threshold expression of *F*^2^ > σ(*F*^2^) is used only for calculating *R*-factors(gt) *etc*. and is not relevant to the choice of reflections for refinement. *R*-factors based on *F*^2^ are statistically about twice as large as those based on *F*, and *R*- factors based on ALL data will be even larger.

### Fractional atomic coordinates and isotropic or equivalent isotropic displacement parameters (Å^2^)

**Table:**

	*x*	*y*	*z*	*U*_iso_*/*U*_eq_	
Cl1	0.90858 (2)	0.20782 (14)	0.56665 (4)	0.0858 (3)	
Cl2	0.96867 (2)	0.10906 (15)	0.90003 (5)	0.0939 (4)	
N1	0.78571 (6)	0.0580 (3)	0.76338 (12)	0.0430 (5)	
N2	0.74565 (5)	0.0416 (3)	0.78172 (11)	0.0449 (5)	
H2	0.7378	0.0201	0.8342	0.054*	
O1	0.83142 (5)	0.1268 (2)	0.64024 (10)	0.0538 (5)	
H1	0.8097	0.1104	0.6635	0.081*	
O2	0.73122 (5)	0.0679 (2)	0.63564 (10)	0.0550 (5)	
O3	0.55192 (6)	0.1318 (3)	0.76671 (15)	0.0877 (7)	
O4	0.28854 (6)	0.0811 (2)	0.04707 (10)	0.0605 (5)	
H18	0.2830	0.0895	−0.0081	0.091*	
H19	0.3050	0.1670	0.0597	0.091*	
C1	0.86185 (7)	0.1190 (3)	0.70224 (14)	0.0445 (6)	
C2	0.90114 (8)	0.1561 (4)	0.67682 (15)	0.0528 (6)	
C3	0.93372 (8)	0.1525 (4)	0.73556 (18)	0.0636 (7)	
H3	0.9597	0.1786	0.7172	0.076*	
C4	0.92734 (8)	0.1095 (4)	0.82283 (17)	0.0596 (7)	
C5	0.88941 (7)	0.0692 (3)	0.85008 (16)	0.0503 (6)	
H5	0.8860	0.0387	0.9090	0.060*	
C6	0.85555 (7)	0.0729 (3)	0.79125 (14)	0.0409 (5)	
C7	0.81465 (7)	0.0354 (3)	0.82230 (14)	0.0397 (5)	
C8	0.80792 (7)	−0.0152 (3)	0.91630 (14)	0.0437 (5)	
H8A	0.8310	−0.0844	0.9394	0.052*	
H8B	0.7840	−0.0916	0.9186	0.052*	
C9	0.80230 (9)	0.1511 (4)	0.97375 (16)	0.0606 (7)	
H9A	0.8275	0.2160	0.9799	0.091*	
H9B	0.7939	0.1141	1.0311	0.091*	
H9C	0.7819	0.2284	0.9466	0.091*	
C10	0.71868 (7)	0.0615 (3)	0.71071 (14)	0.0418 (5)	
C11	0.67535 (7)	0.0788 (3)	0.72990 (14)	0.0422 (5)	
C12	0.66180 (7)	0.1335 (3)	0.81114 (15)	0.0505 (6)	
H12	0.6807	0.1569	0.8570	0.061*	
C13	0.62131 (8)	0.1540 (4)	0.82595 (17)	0.0584 (7)	
H13	0.6130	0.1909	0.8812	0.070*	
C14	0.59297 (8)	0.1198 (4)	0.75892 (19)	0.0593 (7)	
C15	0.60576 (8)	0.0678 (4)	0.67635 (18)	0.0621 (7)	
H15	0.5867	0.0458	0.6306	0.075*	
C16	0.64623 (7)	0.0488 (3)	0.66187 (16)	0.0522 (6)	
H16	0.6545	0.0154	0.6061	0.063*	
C17	0.53716 (10)	0.1785 (6)	0.8521 (2)	0.1014 (12)	
H17A	0.5465	0.0903	0.8950	0.152*	
H17B	0.5080	0.1802	0.8491	0.152*	
H17C	0.5472	0.2962	0.8692	0.152*	

### Atomic displacement parameters (Å^2^)

**Table:**

	*U*^11^	*U*^22^	*U*^33^	*U*^12^	*U*^13^	*U*^23^
Cl1	0.0798 (6)	0.1289 (8)	0.0498 (4)	−0.0187 (5)	0.0173 (4)	−0.0021 (4)
Cl2	0.0387 (4)	0.1699 (10)	0.0722 (5)	0.0028 (4)	−0.0099 (3)	0.0112 (5)
N1	0.0393 (11)	0.0532 (12)	0.0363 (10)	0.0011 (9)	−0.0018 (8)	0.0007 (9)
N2	0.0402 (11)	0.0603 (13)	0.0340 (10)	−0.0005 (9)	−0.0003 (8)	0.0058 (9)
O1	0.0489 (10)	0.0745 (12)	0.0378 (9)	−0.0036 (9)	−0.0019 (7)	0.0026 (8)
O2	0.0523 (10)	0.0788 (12)	0.0337 (9)	0.0002 (9)	−0.0012 (7)	0.0023 (8)
O3	0.0422 (11)	0.1199 (19)	0.1009 (17)	0.0046 (11)	0.0012 (11)	−0.0046 (14)
O4	0.0738 (12)	0.0694 (12)	0.0379 (9)	−0.0159 (9)	−0.0028 (8)	0.0036 (8)
C1	0.0480 (14)	0.0468 (13)	0.0385 (12)	0.0019 (11)	−0.0019 (10)	−0.0035 (10)
C2	0.0504 (15)	0.0648 (16)	0.0439 (13)	0.0003 (12)	0.0082 (11)	−0.0045 (12)
C3	0.0432 (15)	0.085 (2)	0.0629 (17)	0.0017 (13)	0.0084 (13)	−0.0052 (15)
C4	0.0411 (14)	0.082 (2)	0.0554 (15)	0.0071 (13)	−0.0029 (12)	−0.0018 (14)
C5	0.0442 (14)	0.0633 (16)	0.0432 (13)	0.0097 (11)	−0.0017 (11)	0.0019 (11)
C6	0.0415 (13)	0.0423 (13)	0.0388 (12)	0.0050 (10)	−0.0013 (9)	−0.0020 (10)
C7	0.0445 (13)	0.0384 (12)	0.0359 (11)	0.0038 (10)	−0.0023 (10)	−0.0016 (9)
C8	0.0425 (13)	0.0489 (13)	0.0395 (12)	0.0017 (10)	−0.0031 (10)	0.0053 (10)
C9	0.0770 (18)	0.0666 (17)	0.0383 (13)	−0.0001 (14)	0.0055 (12)	−0.0033 (12)
C10	0.0462 (13)	0.0414 (13)	0.0374 (13)	−0.0004 (10)	−0.0044 (10)	0.0002 (10)
C11	0.0426 (13)	0.0409 (12)	0.0425 (12)	−0.0026 (10)	−0.0062 (10)	0.0040 (10)
C12	0.0459 (14)	0.0614 (16)	0.0436 (13)	0.0016 (12)	−0.0049 (11)	−0.0010 (11)
C13	0.0532 (16)	0.0683 (18)	0.0541 (15)	0.0056 (13)	0.0049 (13)	0.0004 (13)
C14	0.0417 (14)	0.0641 (17)	0.0719 (18)	0.0013 (12)	0.0004 (13)	0.0059 (14)
C15	0.0477 (16)	0.0732 (19)	0.0638 (17)	−0.0041 (13)	−0.0179 (13)	−0.0016 (14)
C16	0.0525 (15)	0.0557 (15)	0.0476 (14)	−0.0007 (12)	−0.0073 (11)	−0.0025 (11)
C17	0.0529 (19)	0.139 (3)	0.114 (3)	0.009 (2)	0.0225 (19)	−0.006 (3)

### Geometric parameters (Å, °)

**Table:**

Cl1—C2	1.736 (2)	C7—C8	1.495 (3)
Cl2—C4	1.757 (3)	C8—C9	1.519 (3)
N1—C7	1.289 (3)	C8—H8A	0.9700
N1—N2	1.364 (3)	C8—H8B	0.9700
N2—C10	1.374 (3)	C9—H9A	0.9600
N2—H2	0.8600	C9—H9B	0.9600
O1—C1	1.346 (3)	C9—H9C	0.9600
O1—H1	0.8200	C10—C11	1.473 (3)
O2—C10	1.225 (3)	C11—C12	1.384 (3)
O3—C14	1.364 (3)	C11—C16	1.395 (3)
O3—C17	1.440 (4)	C12—C13	1.369 (3)
O4—H18	0.8499	C12—H12	0.9300
O4—H19	0.8500	C13—C14	1.373 (4)
C1—C2	1.392 (3)	C13—H13	0.9300
C1—C6	1.412 (3)	C14—C15	1.388 (4)
C2—C3	1.365 (3)	C15—C16	1.366 (4)
C3—C4	1.382 (4)	C15—H15	0.9300
C3—H3	0.9300	C16—H16	0.9300
C4—C5	1.364 (3)	C17—H17A	0.9600
C5—C6	1.398 (3)	C17—H17B	0.9600
C5—H5	0.9300	C17—H17C	0.9600
C6—C7	1.470 (3)		
			
C7—N1—N2	122.80 (18)	C8—C9—H9A	109.5
N1—N2—C10	115.55 (18)	C8—C9—H9B	109.5
N1—N2—H2	122.2	H9A—C9—H9B	109.5
C10—N2—H2	122.2	C8—C9—H9C	109.5
C1—O1—H1	109.5	H9A—C9—H9C	109.5
C14—O3—C17	117.7 (2)	H9B—C9—H9C	109.5
H18—O4—H19	106.0	O2—C10—N2	119.8 (2)
O1—C1—C2	118.2 (2)	O2—C10—C11	123.0 (2)
O1—C1—C6	122.7 (2)	N2—C10—C11	117.13 (19)
C2—C1—C6	119.0 (2)	C12—C11—C16	117.7 (2)
C3—C2—C1	122.1 (2)	C12—C11—C10	123.3 (2)
C3—C2—Cl1	119.4 (2)	C16—C11—C10	118.9 (2)
C1—C2—Cl1	118.50 (18)	C13—C12—C11	121.9 (2)
C2—C3—C4	118.7 (2)	C13—C12—H12	119.1
C2—C3—H3	120.6	C11—C12—H12	119.1
C4—C3—H3	120.6	C12—C13—C14	119.7 (2)
C5—C4—C3	121.0 (2)	C12—C13—H13	120.1
C5—C4—Cl2	119.5 (2)	C14—C13—H13	120.1
C3—C4—Cl2	119.5 (2)	O3—C14—C13	124.8 (3)
C4—C5—C6	121.3 (2)	O3—C14—C15	115.7 (2)
C4—C5—H5	119.3	C13—C14—C15	119.5 (2)
C6—C5—H5	119.3	C16—C15—C14	120.4 (2)
C5—C6—C1	117.9 (2)	C16—C15—H15	119.8
C5—C6—C7	120.7 (2)	C14—C15—H15	119.8
C1—C6—C7	121.45 (19)	C15—C16—C11	120.7 (2)
N1—C7—C6	114.57 (19)	C15—C16—H16	119.6
N1—C7—C8	123.8 (2)	C11—C16—H16	119.6
C6—C7—C8	121.57 (18)	O3—C17—H17A	109.5
C7—C8—C9	111.64 (19)	O3—C17—H17B	109.5
C7—C8—H8A	109.3	H17A—C17—H17B	109.5
C9—C8—H8A	109.3	O3—C17—H17C	109.5
C7—C8—H8B	109.3	H17A—C17—H17C	109.5
C9—C8—H8B	109.3	H17B—C17—H17C	109.5
H8A—C8—H8B	108.0		
			
C7—N1—N2—C10	−177.1 (2)	C1—C6—C7—C8	179.4 (2)
O1—C1—C2—C3	179.5 (2)	N1—C7—C8—C9	−88.7 (3)
C6—C1—C2—C3	−1.2 (4)	C6—C7—C8—C9	88.0 (3)
O1—C1—C2—Cl1	−0.7 (3)	N1—N2—C10—O2	9.7 (3)
C6—C1—C2—Cl1	178.64 (18)	N1—N2—C10—C11	−168.88 (19)
C1—C2—C3—C4	0.4 (4)	O2—C10—C11—C12	−157.1 (2)
Cl1—C2—C3—C4	−179.4 (2)	N2—C10—C11—C12	21.4 (3)
C2—C3—C4—C5	0.7 (4)	O2—C10—C11—C16	19.2 (3)
C2—C3—C4—Cl2	−178.6 (2)	N2—C10—C11—C16	−162.3 (2)
C3—C4—C5—C6	−1.1 (4)	C16—C11—C12—C13	1.4 (4)
Cl2—C4—C5—C6	178.2 (2)	C10—C11—C12—C13	177.8 (2)
C4—C5—C6—C1	0.3 (4)	C11—C12—C13—C14	0.0 (4)
C4—C5—C6—C7	−177.8 (2)	C17—O3—C14—C13	−1.9 (4)
O1—C1—C6—C5	−179.9 (2)	C17—O3—C14—C15	177.7 (3)
C2—C1—C6—C5	0.8 (3)	C12—C13—C14—O3	178.4 (2)
O1—C1—C6—C7	−1.8 (3)	C12—C13—C14—C15	−1.1 (4)
C2—C1—C6—C7	178.9 (2)	O3—C14—C15—C16	−178.9 (3)
N2—N1—C7—C6	−175.44 (18)	C13—C14—C15—C16	0.7 (4)
N2—N1—C7—C8	1.4 (3)	C14—C15—C16—C11	0.8 (4)
C5—C6—C7—N1	174.4 (2)	C12—C11—C16—C15	−1.9 (4)
C1—C6—C7—N1	−3.6 (3)	C10—C11—C16—C15	−178.4 (2)
C5—C6—C7—C8	−2.6 (3)		

### Hydrogen-bond geometry (Å, °)

**Table:**

*D*—H···*A*	*D*—H	H···*A*	*D*···*A*	*D*—H···*A*
O1—H1···N1	0.82	1.77	2.494 (2)	146
N2—H2···O4^i^	0.86	2.16	3.004 (2)	168
O4—H18···O2^ii^	0.85	1.97	2.815 (2)	171
O4—H19···O1^iii^	0.85	2.11	2.908 (2)	155

Symmetry codes: (i) −*x*+1, −*y*, −*z*+1; (ii) −*x*+1, *y*, −*z*+1/2; (iii) *x*−1/2, −*y*+1/2, *z*−1/2.
